# High Concentration and Frequent Application of Disinfection Increase the Detection of Methicillin-Resistant *Staphylococcus aureus* Infections in Psychiatric Hospitals During the COVID-19 Pandemic

**DOI:** 10.3389/fmed.2021.722219

**Published:** 2021-10-27

**Authors:** Mi Yang, Yu Feng, Lu Yuan, Huachang Zhao, Shan Gao, Zezhi Li

**Affiliations:** ^1^The Fourth People's Hospital of Chengdu, Chengdu, China; ^2^Center for Pathogen Research, West China Hospital, Sichuan University, Chengdu, China; ^3^University of Electronic Science and Technology of China, Chengdu, China; ^4^Department of Psychiatry, The Affiliated Brain Hospital of Guangzhou Medical University, Guangzhou, China

**Keywords:** MRSA, resistance, disinfection, psychiatric hospital, COVID-19

## Abstract

The tolerance of certain multi-drug resistant bacteria to disinfectants may be promoted while the requirements of environmental disinfection have been raised in the high-risk areas of medical institutions during the COVID-19 pandemic. The current research addressed the mechanisms underlying a sharp increase in the detection of methicillin-resistant *Staphylococcus aureus* (MRSA) observed in a closed-management unit of elderly patients with mental disorders in 2020 as compared with the previous 4 years. We first conducted microbial detection in staff-hand and environment and a molecular epidemiology analysis, rejecting the hypothesis that the MRSA increase was due to an outbreak. Afterward, we turned to disinfectant concentration and frequency of use and analyzed the varied MRSA detection rates with different concentrations and frequencies of disinfection in 2020 and the previous 4 years. The MRSA detection rate increased with elevated concentration and frequency of disinfection, with 1,000 or 500 mg/L two times per day since January in 2020 vs. 500 mg/L 2–3 times per week in 2016–2019. When the disinfectant concentration was reduced from 1,000 to 500 mg/L, the MRSA detection decreased which indicated a modulatory role of disinfectant concentration. With a sustained frequency of disinfection in 2020, the MRSA detection rate was still higher, even after May, than that in the previous years. This suggested that the frequency of disinfection also contributed to the MRSA increase. Overall, the MRSA detection was augmented with the increase in disinfection concentration and frequency during the COVID-19 epidemic, suggesting that highly-concentrated and highly-frequent preventive long-term disinfection is not recommended without risk assessments in psychiatric hospitals.

## Introduction

In December 2020, the first “variant under investigation” of the novel coronavirus was announced in the UK (1). Since then, many countries have reported the rapid spread of new mutant viruses (2, 3). The continuous deterioration of the global epidemic situation has destined the long-term and generalization of this epidemic prevention war. The proposal of “environmental transmission,” a new alternation of transmission, has raised the requirements of environmental disinfection to an unprecedented level. In medical institutions in high-risk areas, using disinfectants to disinfect “object surface and environment” is one of the core means to effectively control the iatrogenic infection (4–7). However, if continuous sterilization with high concentration and high frequency is applied, it may increase disinfectant-resistant strains, especially methicillin-resistant *Staphylococcus aureus* (MRSA) (8, 9).

At the beginning of the COVID-19 epidemic in China, the outbreak of COVID-19 infection in Wuhan Psychiatric Hospital highlighted the differences in management between psychiatric specialist medical institutions and general hospitals, such as fully closed management in hospitalization areas, poor airflow, high personnel density, and long hospitalization cycle wherein the elderly mental patients rarely leave the hospital until they die. Therefore, during the epidemic, our hospital adopted a hierarchical management method for each patient admission unit based on risk assessment and adjusted the concentration and frequency of disinfectant used according to the level of risk. Following the disinfection adjustment, we observed MRSA-related changes in a closed-management unit of elderly mental patients in 2020 and those from the previous 4 years. We further employed whole-genome shotgun sequencing (WGS) of isolated strains to examine the origin of MRSA.

## Materials and Methods

### Preparation and Use of Chlorine-Containing Disinfectants

Effervescent chlorine disinfection tablets were obtained from Chengdu Zhong-Guang Disinfectant Co., Ltd, Chengdu, China (http://www.cdzgxxj.com). Sanitation permission number: Sichuan (Chengdu-Pengzhou) License Number of Healthcare Sterilization [2014] No. 0003; implementation standard number: Q/20193379-8.14. Chlorine disinfection tablets were used to prepare 500 mg/L and 1,000 mg/L disinfectants. Sihuan G-1 disinfectant concentration test paper confirmed the above configuration concentration Beijing, China. The prepared liquid was used for ground and surface disinfection for 30 min and then cleaned with fresh water.

Before January 20, 2020, the concentration and frequency were 500 mg/L 2–3 times per week. From January 20 to May 3, 2020, the concentration and frequency were 1,000 mg/L, two times per day, then after May 4, 500 mg/L, two times per day.

### Source of Strain

*Staphylococcus aureus* was isolated from respiratory tract samples of the geriatric psychiatric unit in the Fourth People's Hospital of Chengdu, Chengdu, China, from 2016 to 2020. The repeated strains of the same patient with pulmonary infection were excluded and only the first detected strains were calculated. Specimen inclusion criteria were as follows: (1) respiratory tract infection and samples were taken before empirical anti-infective therapy was targeted or degraded according to the results of the further etiological examination; (2) in the elderly patients with long-term bedridden or antipsychotic drugs, possible pathogens in sputum were screened when airway secretion increased for the early intervention of the occurrence of respiratory tract infection.

### Bacteria Identification and Drug Sensitivity Test

The identification method was based on the fourth edition of the “National Clinical Laboratory Procedures” for the isolation, culture, and identification of bacteria, using the DL-96 bacteria determination system (Zhuhai DL Biotech. Co., LTD, Zhuhai, China) https://en.medicaldl.com For the corresponding identification/drug sensitivity composite strip for isolation, identification, and drug susceptibility testing, the results were determined according to the NCCLS/CLSI (2015–2019 edition) standard of the American Committee for Clinical Laboratory Standardization. Clinical Test Center of the Ministry of Health supplied *S. aureus* (ATCC25923, ATCC29213), *Escherichia coli* (ATCC25922), *Pseudomonas aeruginosa* (ATCC27853), and *Enterococcus faecalis* (ATCC29212) for identification and drug-sensitive strains test, Beijing, China.

### Whole-Genome Shotgun Sequencing, Genomic Analysis, and Epidemiologic Investigation

The test was approved by the Ethics Committee of the Fourth People's Hospital of Chengdu. The test involved eight patients who provided their written informed consent to participate. Each strain was grown overnight from a single colony in Luria-Bertani (LB) broth, prior to being extracted for DNA using QIAGEN^®^DNeasy Blood & Tissue Kit (Cat No. 69504), Germantown, MD, USA. The layout of the sequencing library was 150 bp paired-end, constructed from genomic DNA of each strain using NEBNext^®^ Ultra™ II DNA Library Prep Kit (Cat No. E7645S) with multiplexing, Ipswich, MA. Indexed samples were pooled and sequenced using the Illumina^®^ HiSeq X Ten system (Illumina, San Diego, USA) by following the instructions of the manufacturer.

Raw reads were filtered by discarding those with adaptor contamination and an average base quality lower than Q20 sequentially using Cutadapt version 3.3 (10). Trimmed reads were then assembled into draft genomes using SPAdes version 3.15.2 (11) under the isolated model, followed by being annotated using Prokka version 1.14.6 (12). Species identification was performed using FastANI version 1.32 (13) by calculating average nucleotide identity (ANI) between the query genome and that of strain ATCC: 12600, which is the type strain of *S. aureus*. Sequence type (ST) and antimicrobial resistances were predicted using Tools MLST version 2.19 (https://github.com/tseemann/mlst) and ABRicate version 1.0.1 (https://github.com/tseemann/abricate), respectively.

A closely related strain of *S. aureus* with a complete genome available in the GenBank, CFSAN007894 (Accession No. CP045866) was selected as the reference genome. Against which, the trimmed reads were mapped and then called for single nucleotide polymorphism (SNP) using pipeline Snippy version 4.6 (https://github.com/tseemann/snippy) with default settings. The pseudo-alignment of genomes was generated by the provided script snippy-core and then fed into Gubbins version 2.4.1 (13) for the detection of recombination regions under the GTRGAMMA model and with a maximum of 50 iterations. A maximum-likelihood tree was then inferred from the recombination-free alignment under the GTR model with gamma-distributed rate variation among sites and tested by 100,000 ultrafast bootstraps using IQ-TREE version 2.1.2 (14). Along with the isolation dates, the emergence of the most recent common ancestor (MRCA) for each clade was estimated in a Bayesian analysis using BactDating version 1.1 (14) under the “mixedcarc” model with 10^7^ iterations of Markov chain Monte Carlo (MCMC).

## Results

### MRSA Positive Detection in Geriatric Psychiatry Department in 2020 and Previous 4 Years

**Figure 1:**
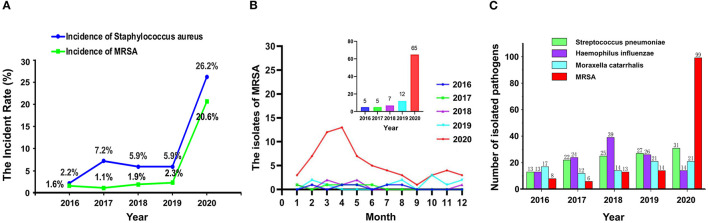
The detection rate of *Staphylococcus aureus* and methicillin-resistant *S. aureus* (MRSA) **(A)**, the isolates of MRSA **(B)**, and the number of pathogenic bacteria **(C)** isolated from respiratory specimens from 2016 to 2020.

In 2020, the detection rate of *S. aureus* and MRSA in lower respiratory tract samples of 180 elderly patients with respiratory tract infection in the psychiatric department was higher than that in the same period of previous years (Figure 1A). In this study, the main types of respiratory tract specimens collected were sputum specimens (including natural sputum expectoration and controllable sputum suction) and throat swabs. Through gram staining of sputum smears, samples with epithelial cells <10 cells/LP and white blood cells >25 cells/LP were regarded as qualified specimens for inoculation and culture. Meanwhile, the recent infectious indicators of patients, such as white blood cell count (WBC), neutrophil percentage (NEU%), high-sensitivity C-reactive protein (hs-CRP), procalcitonin (PCT), among others, were also taken into consideration to determine whether patients had symptoms of infection. If the clinical significance could not be determined by the means, patients were further judged on whether the isolated strains were clinically meaningful pathogens by consulting medical records and imaging data.

### Monthly MRSA Positive Detection in Geriatric Psychiatry Department in 2020 and Previous 4 Years

As shown in Figure 1B, from February to May 2020, the detection of MRSA in lower respiratory tract samples of elderly patients with respiratory tract infection in the psychiatric department was higher than that in the same period of previous years. Even when we changed the disinfectant concentration back to 500 mg/L (but the frequency of use was maintained twice per day) on May 4, 2020, the detection of MRSA was still higher than the same period in previous years.

### The Number of Bacteria Detected in 2020 and the Previous 4 Years

The total numbers of bacteria in the oropharynx or nasopharynx of the infected patients from 2016 to 2020 were 506, 549, 682, 597, and 480, respectively. Figure 1C shows the numbers of different bacteria including MRSA, among others. In 2020, the number of MRSA detected increased rapidly while the other strains changed a little.

### Genetic Characteristics of the Isolated MRSA

**Figure 2:**
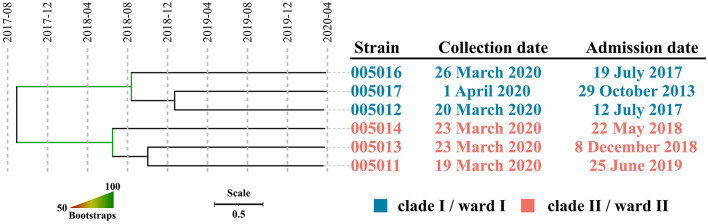
The dated phylogeny of ST5 MRSA isolated around March 2020. Strains within well-separated clades were labeled in either blue (clade I) or red (clade II). Branch supports were colored in gradients.

A total of seven strains were subjected to whole-genome shotgun (WGS) sequencing, producing 1.39 GB clean data (460× depth) on average per sample, which was assembled into draft genomes with an average size of 2,758,102 bp. Gene *mecA* conferring methicillin resistance were found in all the seven strains, with an exception in terms of ST, where strain 005015 belonged to ST338 while all the others (*n* = 6) were identified as ST5. Recombination was found in strain 005014 only and five SNPs within this 800bp region were excluded in the further analysis, leaving a pairwise SNP distance between 5 and 16 for all strains of ST5. Two well-separated clades, which were designated as I and II, were observed in the phylogenetic tree, with an overall nucleotide substitution rate of 4.35 (0.52, 11.7) per genome per year. The emergence of MRCA for clade I, II, and both were estimated to be around August 2018 (June 2013 and December 2019), June 2018 (November 2012 and November 2019), and August 2017 (August 2010 and October 2019), respectively. All the results were supported by that the effective sample size (ESS) of three estimates exceeded 200 (Figure 2).

### Screening for MRSA Colonization or Contamination

Table 1 shows that no MRSA was detected from the environment and surface in the unit and laboratory, and the hands, nasopharynx, and clothing of the staff both in-unit and laboratory.

**Table 1:** Screening of methicillin-resistant *S. aureus* (MRSA) colonization and contamination in the unit and laboratory.

	**Nasopharynx**	**Hands**	**Clothing^a^**	**Air**	**Surface^b^**	**Medical fabrics**
Laboratory	–	None	None	None	None	–
Unit	–	–	–	None	None	None
Staff	None	None	None	–	–	–

a *Clothing includes uniform and daily wearing.*

b *Surface includes equipment, floor, doorknob, and bed unit.*

*None, No MRSA is detected; –, Not available.*

### Monthly MRSA Positive Detection and Concentration of Chlorine-Containing Disinfectants in 2020

**Figure 3:**
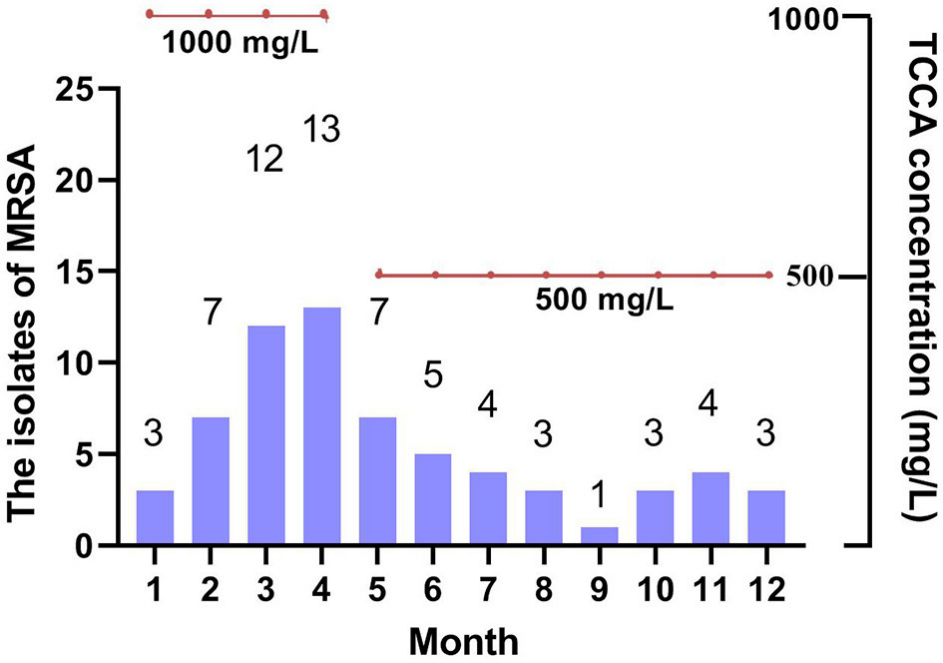
The isolates of MRSA and the concentration of disinfectant in 2020.

As shown in Figure 3, the concentration of chlorine-containing disinfectant decreased to 500 mg/L for the indoor environment and material table of geriatric psychiatric department, the detection number of MRSA began to decline and tended to be stable. The nosocomial infection rate also showed a downward trend.

## Discussion

Overall, with increased concentration and frequency of disinfection (1,000 or 500 mg/L twice per day since January in 2020 vs. 500 mg/L two to three times per week in the previous 4 years), the detection rate of MRSA in sputum samples from the respiratory tract increased. When the disinfectant concentration was reduced from 1,000 to 500 mg/L from May on, the MRSA detection rate decreased, indicating the modulation of the MRSA detection rate by disinfectant concentration. With the sustained frequency of disinfection in 2020, the MRSA detection rate was still higher, even after May, than that in the previous years, suggesting that frequency of disinfection also contributed to the increase of the MRSA detection rate.

Antimicrobial susceptibility test of MRSA from January to April showed that the spectrum of antimicrobial resistance varied among strains, suggesting that increased cases of MRSA infection might not be a consequence of cross-infection in the hospital, and hence it was hypothesized that these infections would be individual cases without a recent epidemiologic linkage.

After exclusion of the duplicated strains, we conducted a subsequent WGS analysis. All seven strains were confirmed to be MRSA members with an ANI well-above the common threshold of 96% when compared with the corresponding type strain and identification of the *mecA* gene, which is consistent with the results of phenotypic assays. Strain 005015, belonging to ST338, represented a separated case of infection, whereas the rest of the six strains of ST5 were considered as related ones due to a small number of SNPs within. However, those of ST5 formed two well-separated clades, suggesting that it was not one but two prevalent lineages. An average nucleotide substitution rate of 4.35 per genome per year was close to that in previous studies, which was estimated to be 4.7 per genome per year (15), indicating that a robust dating of MRCA for each clade could be achieved. The MRCA of clade I, clade II, or both emerged in August 2018, June 2018, and August 2017, respectively, suggesting that the strains responsible for the observed sharp increase of the MRSA infection case might have been circulating in the hospital for almost 3 years and the corresponding patients might be colonized as early as the middle of 2018. Given that these patients had been staying in the ward for over two and a half years on average, it was most likely that they were long-term carriers. Taken together, we concluded that this high detection in such a short time window was not due to a recent or an ongoing outbreak, but two separate lineages of relatively distant MRSA clones colonized the inpatients years ago, which somehow caused infections almost simultaneously. Hence, the trigger probably involved medical workers and disinfectants.

The WGS analysis demonstrated that the MRSA detected was not from an outbreak incidence. To confirm the modulation of the MRSA detection rate by concentration and frequency of disinfection, we excluded exogenous contributors, considering the hospital infection control strategies during the COVID-19 epidemic including no admission of new patients, no visitation for patients, recording of medical staff routines, and prohibition of full-time caregivers from going out. Additionally, there was no environmental modification or equipment replacement in the microbiology room and various quality control standards were met. The MRSA was not detected in the indoor environment, physical surfaces, medical fabrics, hands, skin, nasopharynx, or clothing of the staff in either unit or microbiology room. Infected patients were distributed in non-adjacent beds in different wards and there were no special changes in medication of the patients since the outbreak of the epidemic, ruling out the contribution by infected patients. We also ruled out the possibility of nosocomial outbreaks of iatrogenic infections and laboratory contamination and confirmed the effectiveness of environmental disinfection. One may argue that at the beginning of the pandemic patients wore face masks, which might be a source of respiratory tract MRSA infections. However, after the outbreak of the COVID-19, our hospital prohibited all outsiders from entering the hospital. Given that some patients in the elderly mental health area had problems such as ventilatory disorders and breathing difficulties, each patient did not need to wear a mask. Only the staff and escorts wore masks normally. Therefore, the possibility that the MRSA increase was contributed by the mask-related contaminations was not considered.

The mechanisms underlying the increase in MRSA with elevated concentration and frequency of disinfectant may be that chlorine-containing disinfectants are volatile and can be attached to the mucosa of open channels such as the human respiratory tract for a long time when they are used in a poorly ventilated environment, e.g., a large area with high concentration, leading to the destruction of the microecological balance in the channel. The MRSA, originally planted in the upper respiratory tract of the patient, then develops resistance to chlorine-containing disinfectants and became the dominant bacteria in the sputum samples of the lower respiratory tract. Possible explanations for this were as follows: First, there is an interactive relationship between microbial disinfectant resistance and antimicrobial resistance. Under specific circumstances, disinfectant resistance determined by genes may be associated with antimicrobial resistance via plasmids, transposons, or integrons (16, 17). Previous research has suggested that *S. aureus* contains a variety of antibiotic and disinfectant-resistant plasmids, which relate to each other in a certain way (18–20). Second, local low concentration (chlorine-containing disinfectant attached to the body cavity after volatilization, showing a low concentration) may repeatedly stimulate the colonization of MRSA, leading to the formation of its biofilm. The biofilm blocks the entrance of active disinfectant components, thus protecting the bacteria in the biofilm from reaction with the disinfectant (21–24) and resulting in disinfectant resistance of *S. aureus*. Third, many by-products decomposed during the use of disinfectants had been shown to induce mutations in organisms, which contributed to the emergence of a large number of drug-resistant microorganisms (25–27). Fourth, bacteria might trigger common cellular reactions to counteract the effects of disinfectants and antibiotics and there may be co-selection or co-metastasis of tolerance.

The use of high-concentration chlorine-containing disinfectants has disrupted the micro ecological balance of patients, leading to the increased detection rate of MRSA. The WGS analysis found that the detected strains were all homologous except for newly admitted patients, which reveals the potential risk of nosocomial infection in the hospital. First, MRSA patients were bedridden for a long time and had a history of repeated anti-pulmonary infection treatment. There are still problems in the standardized use of antibiotics due to insufficient types of antibiotics available in psychiatric hospitals, resulting in a great risk of production and colonization of multi-resistant strains. Therefore, the elderly patients with almost no turnover in psychiatric hospitals should be actively screened for multiple drug-resistant bacteria on admission or regularly, to achieve early intervention for carriers of multi-drug-resistant bacteria, and at the same time, the use of antibiotics should be standardized to avoid the formation and colonization of drug-resistant strains and reduce the chance of transmission of multi-drug-resistant bacteria in the hospital. Regular decolonization such as mouthwash, baths, or showers with chlorhexidine, and nasal mupirocin could effectively reduce the risk of MRSA infection (28). Second, based on the traditional risk assessment models including the severity of patients and the difficulty of environmental control in institutions such as psychiatric specialties where the average hospital stay is more than 50 days, the sterilization methods of using a single disinfectant with different concentrations and different frequencies should be discussed. We could combine microorganisms with the results of active screening and environmental sampling mentioned above, select the types of disinfectants that match the indications and employ the appropriate concentration and frequency under the guidance of the pharmacy specialist, to replace the disinfectants from the regular reservoir. Thus, we could gradually construct “differential implementation plans of environmental disinfection” in the hospital. This means that different types of disinfectants should be determined for different ward units according to the common bacteria detected in the region and different disinfectant concentrations should be applied according to different sub-regional needs.

It is noteworthy that only one psychiatric hospital was involved, and no other ones have been under investigation. Without detecting similar MRSA infection outbreak cases in other psychiatric hospitals, it was hard to decide whether our observation is a common phenomenon. Further work may include multi-center data to verify the present findings. The inclusion of only seven isolates in the WGS analysis was another limitation in the present study. At the beginning of the MRSA infection outbreak, we assessed potential causes in terms of environment and person, failing to consider WGS in time. Instead of gene sequencing, only the phenotype of the antimicrobial sensitivity test was used to preliminarily determine whether the MRSA were non-homologous strains. It was not until the detection of MRSA continued to increase and the use of low-concentration chlorinated preparations was started at the same time that strains were purposefully preserved for subsequent systematic analysis, resulting in the failure of preserving most of the early detected strains in time. There were also technical reasons that contributed to the small number of isolates sequenced. Future work should consider preserving strains in time for follow-up epidemiological research when similar issues are encountered.

Above all, the dual control of disinfectants and antibiotics should be implemented via the control and management of iatrogenic infection in elderly psychiatric inpatients. The existence of anti-disinfectant strains is a potential hazard for hospital disinfection failure, especially when strains with both a disinfectant and antibiotic resistance characteristics become dominant. It increases the difficulty in hospital infection control (29, 30). Therefore, we do not recommend the use of high concentrations of chlorine-containing disinfectants (e.g., higher than 1,000 mg/L) for preventive disinfection (n.d.), which can also be instructive and applicable to the normalized sterilization management of epidemic situations in similar long-term care institutions for the elderly. Considering that recurrent infections of MRSA are common (31), we examined the infection cases from January to August in 2021 and found that with the use of disinfectant turned back to normal conditions (500 mg/L, two times per day) the MRSA infection frequency has been keeping low. Future research may investigate the tolerance mechanism of MRSA to chlorine-containing disinfectants in animal model experiments.

## Data Availability Statement

The original contributions presented in the study are publicly available. This data can be found at: National Center for Biotechnology Information (NCBI) BioProject database under accession number PRJNA450214.

## Ethics Statement

The studies involving human participants were reviewed and approved by the Ethics Committee of the Fourth People's Hospital of Chengdu. The patients/participants provided their written informed consent to participate in this study.

## Author Contributions

MY, YF, and ZL contributed to the literature search and conception of the study. MY, LY, and HZ contributed to the acquisition and analysis of the data. MY, YF, SG, and ZL interpreted results. MY, SG, and ZL wrote the study. All the authors discussed the results and commented on the manuscript.

## Funding

This study was supported by the National Natural Science Foundation of China (Grant Nos. 61806042 and 62073058) and the Special Research Project for the novel coronavirus pneumonia funded by the Chengdu Science and Technology Bureau (Grant No. 2020-YF05-00171-SN).

## Conflict of Interest

The authors declare that the research was conducted in the absence of any commercial or financial relationships that could be construed as a potential conflict of interest.

## Publisher's Note

All claims expressed in this article are solely those of the authors and do not necessarily represent those of their affiliated organizations, or those of the publisher, the editors and the reviewers. Any product that may be evaluated in this article, or claim that may be made by its manufacturer, is not guaranteed or endorsed by the publisher.
